# Anorexia Nervosa in the Gazes of Primary Healthcare Practitioners

**DOI:** 10.1192/j.eurpsy.2023.1807

**Published:** 2023-07-19

**Authors:** Z. O. Moldokulova, E. Molchanova

**Affiliations:** Psychology, American University of Central Asia, Bishkek, Kyrgyzstan

## Abstract

**Introduction:**

Eating Disorders (EDs) as a public health concern were explored by numerous studies, where attention was drawn to further exploration of Anorexia Nervosa (AN) in non-western countries due to the existing gap in the area (Cummins et al., 2005; Javier & Belgrave, 2019). The studies emphasized the importance of exploring the perceptions and attitudes of healthcare practitioners in order to improve the quality of medical care for people who have AN (Atti et al., 2020; Colmsee et al., 2021; Costa-Val et al., 2019; Ghaderi et al., 2020; Jafar & Morgan 2021; Kohrt et al., 2020; Reas et al., 2021). Little research related to EDs in Central Asia has been found among previous literature. This study contributes to further research in this area taking into account societal and cultural specifics existing in Kyrgyzstan.

**Objectives:**

Healthcare practitioners are accessible for the majority of the population of Bishkek through the local primary healthcare (PHC) structure. Therefore, the perceptions of the local PHC workers were explored within the present study. The aim of the current research was to explore how primary healthcare practitioners perceive AN and people who have AN in Bishkek, Kyrgyzstan.

**Methods:**

Six semi-structured interviews with six PHC workers who currently provide medical care for the local population were conducted in accordance with the Interpretative phenomenological analysis (IPA). The results were discussed in frames of the Social Constructionism theory.

**Results:**

The interviews uncovered major themes which show how the participants perceive AN and people with AN. According to the participants, AN develops due to stress. Families of AN patients carry the heavy burden of the disorder. The participants described how local and western socio-cultural standards influence the development of AN and seeking treatment process. AN patients do not want to be treated due to the stigmatization of mental health area within the local population and self-stigmatization of mental health disorders. However, the participants themselves have both positive and negative attitudes towards the mental healthcare.

**Conclusions:**

The participants represent local societal perceptions and attitudes related to EDs and AN among medical professionals in accordance with the social constructionism theory. Overall, the PHC physicians in Bishkek have positive attitudes towards AN patients. They feel compassionate towards people who need their professional help. However, the participants feel like they are not able to provide the appropriate medical help for the population with AN due to the way PHC structure in Bishkek, Kyrgyzstan functions. Local PHC practitioners do not receive enough resources to provide medical care for people with mental health issues in general. The important discourse uncovered within the present study is the attention brought to the local PHC structure.

**Disclosure of Interest:**

None Declared

